# Editorial: Promoting compliance with biosecurity in animal production

**DOI:** 10.3389/fvets.2023.1215433

**Published:** 2023-06-01

**Authors:** Julia M. Smith, Claude Saegerman, Jean-Pierre Vaillancourt

**Affiliations:** ^1^Department of Animal and Veterinary Sciences, University of Vermont, Burlington, VT, United States; ^2^Department of Infectious and Parasitic Diseases, University of Liege Veterinary Medicine, Liege, Belgium; ^3^Department of Clinical Sciences, University of Montreal, St-Hyacinthe, QC, Canada

**Keywords:** livestock, biosecurity, animal biosecurity, compliance, culture, change, communication

## Introduction

Globally, livestock and poultry industries are facing the emergence, re-emergence and re-circulation of highly contagious infectious diseases. Entry of disease into herds or flocks may be due to lack of biosecurity protocols, use of ineffective protocols or lack of compliance with protocols. Imperfect compliance can negate or nullify the efforts of others and expose animals to disease threats with consequences to premises, production systems and countries locally or globally.

Effective measures to prevent infectious disease spread have been known for centuries. Yet we are still struggling to apply these today on multiple levels in animal production. In practice, stumbling blocks to developing and carrying out biosecurity plans are encountered: These can be found at the operational level where people work on the premises, at the tactical level where owners or managers make decisions about protocols and at the strategic level where industry or other regulatory policies come into play. The path forward begins with understanding why management systems and staff are failing to implement effective biosecurity and how to influence those involved to do better. Our understanding of what influences human behavior in the context of animal health and biosecurity has been bolstered in recent decades by transdisciplinary efforts among veterinary scientists and economists, communication specialists, socio-anthropologists, education specialists, and psychologists (1–5). This understanding can be used to engineer solutions physically, technologically, psychologically, and socially. The goal of this Research Topic is to regroup current knowledge on what works and does not work, why that is and how we can go forward in order to have immediate and lasting practical solutions to the problem of infectious disease spread in animal production.

## Organization of this Research Topic

This Research Topic includes seven papers relevant to mitigating specific diseases of swine and poultry and one paper by Humblet and Saegerman on compliance with disease control practices in a veterinary teaching hospital setting. Larsen et al. look at the relation between knowledge and biosecurity practices. Liu et al. look at communication and compliance with biosecurity protocols, specifically in response to messages translated for audiences for whom English is not their native language, whereas Racicot et al. look at the impact of a monitoring system on compliance. Havas et al. take a preliminary look at the effectiveness of investing in tactical biosecurity practices in a system of sow farms. Finally, a set of papers (Koliba et al.; Clark et al.; Bucini et al.) discusses and illustrates taking a systems approach to understanding the impact of human behaviors on disease spread.

Humblet and Saegerman audited compliance with biosecurity standard operating procedures for a specific sector/activity in a veterinary college. The identification of criteria needing improvement helps prioritize actions to be implemented and raise awareness among people concerned. Indeed, regular internal auditing is an essential part of a living biosecurity plan.

Larsen et al. conducted a survey of *Salmonella* prevalence in backyard poultry and what owners know and do in terms of biosecurity. This approach connects risk perception with human behavior while simultaneously collecting quantitative risk information.

Havas et al. report a case study of biosecurity and PRRS in sow units of a swine production system with 67 sow farms. The biosecurity interventions of interest in this study included feed additives (i.e., mitigants), high efficiency particulate absorbance (HEPA) air filtration, and location of farm in terms of swine density by state. These are examples of a tactical, mostly technological, approach to disease mitigation.

Technology meets human behavior in the paper by Racicot et al. Calling back to the theme of auditing studied by Humblet and Saegerman, this study endeavored to demonstrate whether monitoring and real-time feedback improve compliance. The importance of pilot testing such schemes is underscored by the realization that human behavior is not always predictable.

The response of human behavior to instructions is also inconsistent. Liu et al. honed in on the differential responses to messages translated into different languages. While not able to completely segregate the effect of language and culture, these considerations are very relevant to animal industries where employees do not all share the same cultural background.

Koliba et al. describe how a series of experimental games and simulations have examined human decision-making regarding animal biosecurity at the operational, tactical and strategic levels. Collaborators with the University of Vermont's Social Ecological Gaming and Simulation laboratory have spearheaded a novel and transdisciplinary approach to understanding how decisions made on individual production units relate to disease spread at broader geographic scales. The power of this approach is that it enables testing of various approaches (e.g., messages, incentives, or other policy tools) for response at the operational level and simulating the effect across the pork supply chain.

The paper by Clark et al. describes results of a specific experimental game designed to identify behaviors related to risk assessment and disease mitigation. By utilizing a payment structure pegged to performance in the “game,” MTurk participants were incentivized to actively engage in the simulation and not just “click through.” The analysis of data from over 1,200 participants identified three clusters of responses categorized as risk tolerant, risk averse, and opportunist. These response types can then be incorporated into agent based models representing these heterogeneous positions in relation to biosecurity implementation.

Bucini et al. used such an agent-based model to explore how behaviors affect disease spread through the animal protein supply chain. Created in the context of PEDv, the model builds on the results of previous experimental games focused on compliance (6), risk attitude (7, 8), and learning (9).

## Conclusion

This Research Topic highlights a variety of innovative contributions to inform practical management options for better promotion and compliance with biosecurity in animal production. Further work is needed on drivers of behavior, the role of cross-cultural communication, the intersection of technologies and behaviors, the effectiveness of training programs, and appropriate monitoring to facilitate compliance with biosecurity protocols to protect animal health. In addition, better understanding of the how decision-making at different levels interacts with the dynamics of disease spread will support policy innovations.

## Author contributions

All authors listed have made a substantial, direct, and intellectual contribution to the work and approved it for publication.
